# Fatty Acid Modulation of the Endocannabinoid System and the Effect on Food Intake and Metabolism

**DOI:** 10.1155/2013/361895

**Published:** 2013-05-26

**Authors:** Shaan S. Naughton, Michael L. Mathai, Deanne H. Hryciw, Andrew J. McAinch

**Affiliations:** ^1^Biomedical and Lifestyle Diseases Unit, College of Health and Biomedicine, Victoria University, P.O. Box 14428, Melbourne, VIC 8001, Australia; ^2^Florey Neuroscience Institutes, The University of Melbourne, Melbourne, VIC 3010, Australia; ^3^Department of Physiology, The University of Melbourne, Melbourne, VIC 3010, Australia

## Abstract

Endocannabinoids and their G-protein coupled receptors (GPCR) are a current research focus in the area of obesity due to the system's role in food intake and glucose and lipid metabolism. Importantly, overweight and obese individuals often have higher circulating levels of the arachidonic acid-derived endocannabinoids anandamide (AEA) and 2-arachidonoyl glycerol (2-AG) and an altered pattern of receptor expression. Consequently, this leads to an increase in orexigenic stimuli, changes in fatty acid synthesis, insulin sensitivity, and glucose utilisation, with preferential energy storage in adipose tissue. As endocannabinoids are products of dietary fats, modification of dietary intake may modulate their levels, with eicosapentaenoic and docosahexaenoic acid based endocannabinoids being able to displace arachidonic acid from cell membranes, reducing AEA and 2-AG production. Similarly, oleoyl ethanolamide, a product of oleic acid, induces satiety, decreases circulating fatty acid concentrations, increases the capacity for **β**-oxidation, and is capable of inhibiting the action of AEA and 2-AG in adipose tissue. Thus, understanding how dietary fats alter endocannabinoid system activity is a pertinent area of research due to public health messages promoting a shift towards plant-derived fats, which are rich sources of AEA and 2-AG precursor fatty acids, possibly encouraging excessive energy intake and weight gain.

## 1. Introduction

Overweight and obesity rates are reaching epidemic proportions worldwide [1] and as such are considered two of the most important medical conditions of current day, due not only to the effect on general health and further disease development (such as type II diabetes mellitus (T2D) and cardiovascular disease) but also to the financial burden placed on the healthcare system [2]. For excess body weight to develop a positive energy balance is required, either through insufficient energy expenditure or excessive energy intake [3]. Populations in developed countries are currently consuming more than 30% of their energy intake in the form of energy dense fats, a dietary pattern often coupled with an excessive intake of highly palatable, sugar-rich processed, and convenience foods, promoting accumulation of excess body weight [4, 5]. Food intake is influenced greatly by appetite, with homeostatic food intake being in response to an increase in appetite triggered by a decrease in energy availability; conversely, hedonic food intake is triggered by appetite in response to endogenous and exogenous stimuli and often occurs in satiated or postprandial states [6]. Both homeostatic ingestion and hedonic ingestion result in an increase in circulating neurotransmitters, hormones, and glucocorticoids which have the potential to regulate the activity of a number of G-protein coupled receptors (GPCR), including the cannabinoid receptors [7, 8].

## 2. The Endocannabinoid System 

The endocannabinoid system is implicated in both homeostatic and hedonic food intakes [9], with activation of the system resulting in an increase in hunger [10, 11]. Specifically, anandamide (AEA) and 2-arachidonoyl glycerol (2-AG), which are derivatives of arachidonic acid (AA) [7, 12, 13], bind to the main two receptors, cannabinoid receptor 1 (CB_1_) and cannabinoid receptor 2 (CB_2_), leading to activation of pathways to initiate food intake in the limbic system [14], hypothalamus [15, 16] and hindbrain [17]. CB_1_ and CB_2_ belong to the GPCR class of receptors, generally signalling through G_i/o_ proteins, though chronic low level stimulation triggers a shift to signalling through G_s_ proteins [18]. AEA is a ligand for CB_1_ [19], with a reduced affinity for CB_2_ [12], whereas 2-AG binds to both receptors [20–22]. Though there are structural differences between the glycerol-based and the *N*-acylethanolamine- (NAE-) based endocannabinoids, they share common receptor pathways and functions, with all compounds involved in appetite and modulation of metabolism signalling through GPCR or altering GPCR signalling [21, 23, 24].

**Figure 1:**
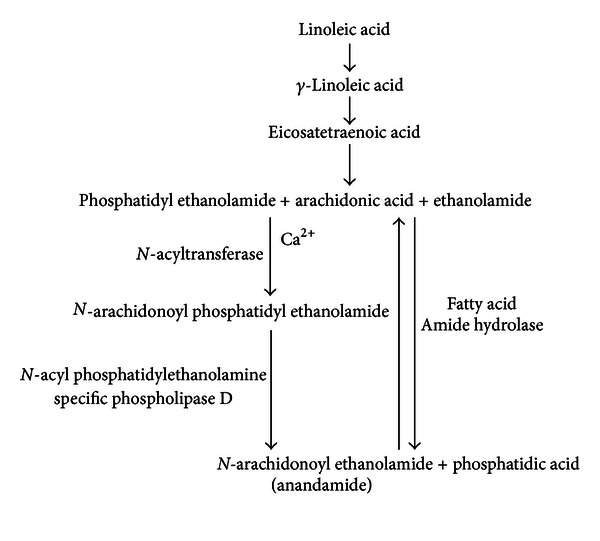
Anandamide synthesis pathway. Pathways involved in the synthesis of anandamide from dietary linoleic acid and arachidonic acid, via the addition of either phosphatidyl ethanolamide or ethanolamide, the latter also resulting in phosphatidic acid production. Adapted from the works of Salem et al. [31], Sugiura [32], Cravatt et al. [33], Cadas et al. [34], and Okamoto et al. [35].

**Figure 2:**
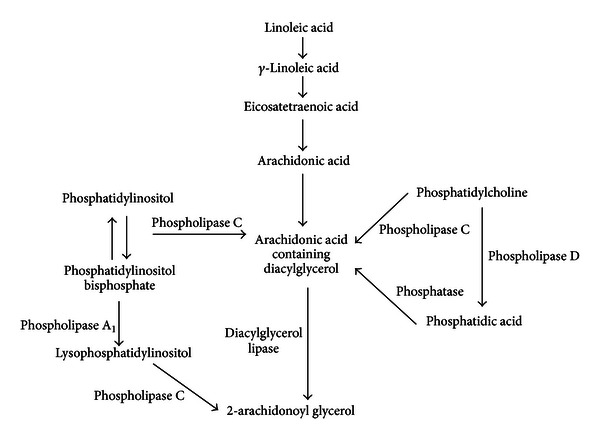
2-arachidonoyl glycerol synthesis pathway. Pathways involved in the synthesis of 2-arachidonoyl glycerol from dietary linoleic acid and arachidonic acid, as well as from phosphatidylinositol, phosphatidylinositol bisphosphate, phosphatidylcholine and phosphatidic acid. Adapted from the works of Salem et al. [31], Venance et al. [36], Kondo et al. [37], and Tsutsumi et al. [38].

Endocannabinoids are products of dietary fatty acids (FA) and were originally thought to be generated on demand [25–27], though it is now known that AEA can be stored in intracellular lipid droplets [28]. As such, modulation of cannabinoid receptor function can occur via modification of dietary FA intake. Current dietary guidelines recommend a shift away from animal-derived fats in favour of plant fats, in an effort to reduce saturated fat intake and cardiovascular disease risk, which has resulted in an increased intake of polyunsaturated fatty acids (PUFA), especially that of linoleic acid [29, 30]. Linoleic acid is easily converted by the human body to AA via *γ*-linoleic acid and eicosatetraenoic acid, a pathway dependent on the actions of two desaturases and one elongase [31]. AA can then be converted to AEA via several pathways as shown in Figure 1, including the condensation of AA and ethanolamide due to the reverse activity of fatty acid amide hydrolase (FAAH), as reviewed by Sugiura [32]. As FAAH is also the main enzyme responsible for AEA breakdown, its action is also capable of decreasing cannabinoid receptor activation through a reduction in the availability of agonists [33]. Another anabolic pathway involves the biosynthesis of *N*-arachidonoyl phosphatidylethanolamine (NAPE) resulting from the transfer of *sn-*1 position AA from phospholipids to phosphatidylethanolamine by a Ca^2+^, dependent *N*-acyltransferase. NAPE can then be converted to AEA and phosphatidic acid by *N*-acyl phosphatidylethanolamine specific phospholipase D (NAPE-PLD) and is believed to be the major source of AEA [34, 35]. Similarly there are several pathways through which 2-AG can be synthesised, as shown in Figure 2. One of these pathways involves the conversion of diacylglycerol to 2-AG via diacylglycerol lipase, with diacylglycerol being produced from phosphatidylinositol, phosphatidylcholine, or phosphatidic acid, with the latter two being synthesised by phospholipase C and phosphatases, respectively [36, 37]. Phospholipase C is also capable of converting phosphatidylinositol bisphosphate to diacylglycerol and lysophosphatidylinositol to 2-AG, though this requires a specific phospholipase C isoform [38]. As phospholipase C (which is a key enzyme in 2-AG synthesis) is part of downstream GPCR signalling, producing diacylglycerol, it has been found that other GPCRs, including the angiotensin AT_1_ receptor, are capable of paracrine transactivation of CB_1_ [39, 40], which may indicate that 2-AG synthesis can be influenced by activation of coexpressed GPCRs.

With dietary fats being the only source of FA required for synthesis of endocannabinoids, it is possible that what is being consumed is capable of modulating circulating endocannabinoid levels, thus influencing GPCR signalling in an acute time frame and affecting appetite and subsequent food intake. Also, specific FA, such as AA, are favourably incorporated into phospholipids as opposed to triglycerides [41], further affecting their fate in regard to endocannabinoid synthesis due to their cellular location; however, the role of storage in the acute effects of dietary fats and later endocannabinoid synthesis requires further investigation. 

## 3. Overweight, Obesity, and the Endocannabinoid System

Clear associations between body weight and modulation of the endocannabinoid system have been found. The most common of these is that circulating 2-AG levels are significantly increased in obese individuals compared to lean controls [42, 43]. Furthermore, there are positive correlations between 2-AG and body mass index (BMI) (kg/m^2^) [44], waist circumference, and intra-abdominal adiposity [43, 45]. This may be due to the activity of monoacylglycerol lipase (MGL), which primarily degrades 2-AG, not increasing with BMI [43, 45], though expression of FAAH, which is also capable of breaking down 2-AG [46], does increase with BMI [47]. This increase in 2-AG may also be a result of increased diacylglycerol lipase in obesity, which has been demonstrated in both animal [48, 49] and human adipocytes [50, 51], though this may be site specific [50] and influenced by dietary composition [52]. An increase in FAAH has been found to result in decreased subcutaneous adipose tissue 2-AG levels in obese subjects when compared to lean controls [53], with 2-AG also being positively correlated with visceral CB_1_ gene expression [42]. Body weight has also been found to influence cannabinoid receptor expression, with significant correlations found between CB_1_ expression and BMI [54], percentage body fat [42], and the presence of the metabolic syndrome (independent of BMI) [54]. A correlation in obese individuals has also been found between circulating insulin and increased visceral adipose tissue CB_1_ expression, compounded by the presence of the metabolic syndrome, perpetuating visceral lipogenesis due to the role of CB_1_ in promoting energy storage in adipose tissue [54].

Overweight and obese individuals often have a dysregulation of the endocannabinoid system in peripheral tissues, affecting glucose and lipid metabolism [50, 55, 56]. Demonstrating this, a study using paired adipose tissue samples found greater CB_1_ mRNA expression in visceral adipose tissue than subcutaneous, with a negative correlation between visceral fat mass and FAAH mRNA expression [42]. Similarly, other studies have found that genes involved in 2-AG, CB_1_, and MGL synthesis are downregulated in gluteal and upregulated in abdominal adipose tissue of obese individuals [50, 55]. As activation of CB_1_ results in increased glucose uptake [57], this may indicate preferential energy storage in abdominal adipose tissue. Supporting this, glucose uptake in adipocytes is increased by treatment with 2-AG [58] or AEA (which increased glucose uptake 2-fold) [59] with insulin resistant adipocytes from obese mice showing increased expression of endocannabinoid synthesising enzymes and decreased degrading enzymes [48]. Moreover, CB_1_ expression is increased in adipocytes during differentiation, as is peroxisome proliferator-activated receptor *γ* (PPAR*γ*) expression which promotes lipid uptake and adipogenesis [50, 60], both of which are perpetuated by hyperglycaemic conditions [61]. Also, agonism of CB_1_ with either WIN 55212-2 [62] or HU-210 [61] in cultured adipocytes increases PPAR*γ* expression, lipid droplet formation, and adipocyte differentiation [61, 62]. PPAR*γ* activity increases adipocyte differentiation. During differentiation, CB_1_ expression is increased, and subsequent increases in CB_1_ activity increase PPAR*γ* expression. Thus, chronic stimulation of CB_1_ may lead to a cycle of increased adipocyte differentiation and thus further CB_1_ expression. In contrast, in normal weight wild-type mice, agonism of CB_1_ with HU-210 significantly reduces glucose uptake from skeletal muscle fibres (due to decreased serine/threonine-specific protein kinase (Akt) phosphorylation) curtailing whole body uptake [63]. Similarly CB_1_ agonism with arachidonoyl-2-chloroethylamide in lean rat muscle significantly reduces both basal and insulin stimulated glucose uptake [64]. 

## 4. Acute Modulation of the Endocannabinoid System

Research on acute modulation of the endocannabinoid system by dietary intake in humans is extremely limited and has generally focused on macronutrient ratios rather than specific FA intakes. Gatta-Cherifi et al. (2011) and Matias et al. (2006) have assessed the effect of food intake on acute concentrations of endocannabinoids [61, 65]. Gatta-Cherifi et al. (2011) compared nondiabetic insulin resistant obese subjects to healthy normal weight participants (though not age or gender matched [65]), whereas Matias et al. (2006) compared healthy participants (though the average BMI was 28.6 ± 1.9, classifying them as overweight according to the World Health Organisation [61]) to obese diabetic hyperglycaemic subjects. These studies tested different meal compositions with Gatta-Cherifi et al. (2011) using a meal comprising 35% of energy from lipids, 45% carbohydrate, and 20% protein [65], while Matias et al. (2006) utilised a high fat meal (44.15% of energy from lipids, 39.25% carbohydrate, and 16.6% protein [61]). Both studies showed obese subjects to have increased fasting plasma AEA and 2-AG concentrations, indicating potential chronic cannabinoid receptor overstimulation [61, 65], with Gatta-Cherifi et al. (2011) finding positive correlations between AEA/2-AG levels and both BMI and waist circumference [65]. A positive correlation was also found between AEA and insulin levels in the obese group [65], demonstrating CB_1_ overactivity in insulin resistant individuals. This study also found that in the hour after meal consumption AEA levels decreased only in lean subjects, indicating greater orexigenic stimulus in the obese individuals [65], possibly leading to short term hedonic food intake and therefore excess energy intake. Meal consumption by normoglycaemic participants in the Matias et al. (2006) study resulted in transient hyperglycaemia, triggering significant insulin level increases and a concomitant reduction in AEA levels [61] with results from the same study (assessing saliva as opposed to plasma) finding significant reductions in oleoyl ethanolamide (OEA) [66]. 

A study by Monteleone et al. (2012) investigated the acute (2 hour) influence of hedonic eating in healthy weight satiated individuals with two different meals, one which subjects found extremely palatable and one with the same energy density and nutrient composition which was not considered palatable [9]. One major strength of this study was that participants consumed as much of the palatable food as they wanted in a 10-minute period and were then given the same volume of the nonpalatable meal to eat in the same time frame during a second session, removing the variables of time taken to eat and amounts of ingested nutrients. In the 120 minutes after consumption there were no significant differences between the two meals in appetite or satiety scores. Both meals triggered significant AEA and OEA decreases, though the palatable meal resulted in significantly increased plasma 2-AG 2 hours postprandially, accompanied by a significant rise in ghrelin [9]. Supporting this finding, a study assessing 2-AG changes in mice in response to a palatable high fat diet found that levels were increased when compared to control fed animals, which further induced a preference for the high fat diet [67]. This may demonstrate the cyclic nature between hedonic eating, or the intake of pleasurable foods, and increases in 2-AG and orexigenic cannabinoid receptor stimulation. 

One study investigating the effect of ethanol on endocannabinoid levels involved the consumption of a test meal (21% of energy from lipids, 62.9% carbohydrates, and 16.1% protein) in a group of 19 lean premenopausal women [68]. This is the only research thus far, to the author's knowledge, to demonstrate a correlation between serum FA and their respective endocannabinoids (2-AG was not measured in this study) [68], though this study was performed in a nonfasting cohort. This study found the strongest correlation between OEA and its precursor, oleic acid, though a correlation was also found between AA and AEA [68]. Furthermore, a correlation was found between circulating AEA levels and serum total free FA and BMI over the three-hour monitoring period [68], though unfortunately relationships between consumed FA, serum FA, and circulating endocannabinoids were not investigated. With the subjects in this study being lean and having normal blood lipid profiles, this demonstrates that without the modulation of the endocannabinoid system by obesity, a high fat meal may still be capable of increasing acute circulating AEA and therefore CB_1_ stimulation, possibly perpetuating further food intake, preferential adipose tissue energy storage, and adipogenesis.

## 5. Influence of High Fat Diets on Endocannabinoid Synthesis

Worldwide, high fat diets (~40% of energy) are increasing in prevalence due to the low cost of fats and also due to their palatability [69, 70]. High fat diets are capable of modulating levels of endocannabinoids regardless of their FA composition [71–73]. In animals, high fat diets trigger binge eating patterns [67] and result in significantly increased intestinal motility [56] and AEA and 2-AG levels [74, 75] possibly increasing cannabinoid receptor stimulation. High fat diets also result in increased FA synthesis which is in part due to chronic CB_1_ activation increasing expression of the lipogenic transcription factor sterol regulatory element-binding protein-1c (SREBP-1c), triggering greater production of acetyl coenzyme-A carboxylase-1 and fatty acid synthase [75]. Increased levels of AEA and 2-AG in response to high fat diets in animals have been found to occur due to decreased MGL and FAAH activity and increased NAPE-PLD action [76], which occurs irrespective of ingestion, as demonstrated by sham feeding studies [73, 77]. Compounding this, a high fat diet when part of both hypercaloric and isocaloric diets has been found to decrease OEA levels independent of NAPE-PLD activity, further promoting food intake [72]. 

## 6. The Effect of Dietary Saturated Fat Intake on Endocannabinoid Production

Research into the effect of saturated fats on the endocannabinoid system is extremely limited with the exception of palmitic and stearic acids. One study, however, using a pharmacological dose of stearoyl ethanolamide, has demonstrated a reduction in food intake in starved mice when administered intravenously [78]. The levels of the palmitic acid based palmitoyl ethanolamide (PEA) are, however, not believed to be affected by starvation/refeeding or greatly affected by the intake of any specific nutrients [79]. However, levels of PEA have been found to be reduced in rat brain, liver, and small intestine when eicosapentaenoic acid (EPA) and docosahexaenoic acid (DHA) are administered orally in pharmacological quantities [80]. To the authors' knowledge only one study has found PEA to modulate appetite [81], though it has been demonstrated to be capable of activating PPAR*α* [82] and is also able to bind to GPR55 [83]; however, further research is required to confirm these observations.

One study has investigated lauroyl ethanolamide, from the precursor lauric acid, finding it capable of stopping AEA synthesis in cultured rat basophilic leukaemia and glioma cells [84]. This is supported by human studies which have found that intraduodenal infusion of lauric acid decreases appetite and energy intake [85, 86] with it having a greater effect on appetite and subsequent energy intake than an oleic acid infusion of the same load [87], although these studies did not investigate the involvement of the endocannabinoid system. 

## 7. The Effect of Dietary Oleic Acid on Endocannabinoid Production

The main monounsaturated fatty acid (MUFA) to be investigated in relation to endocannabinoid synthesis has been oleic acid, the primary FA in olive oil [72]. This is due to oleic acid being the precursor for OEA, with synthesis being dependent on the membrane FA transporter CD36 [88]. OEA has been found to reduce levels of ghrelin and neuropeptide YY [89] and subsequently food intake [90] in starved rats when administered intravenously [91, 92]. Oral administration as part of a high fat diet in mice results in increased FAAH and adiponectin gene expression, resulting in decreased food intake and adipose tissue mass indicative of a reduction in CB_1_ agonism [27, 93]. Oral administration also decreases hepatocyte lipid content, serum triglycerides and cholesterol [94], gastric emptying, and intestinal motility [76]. Furthermore, OEA increases satiety through activation of PPAR*α* [95] as well as increasing PPAR*α* regulated gene expression, including that of PPAR*α*, fatty acid translocase, fatty acid transport protein 1 [96], liver fatty-acid binding protein, and uncoupling protein-2 [94]. This therefore increases *β*-oxidation capacity and decreases circulating FA [97, 98] which may be precursors for endocannabinoid synthesis or contribute to decreased glucose uptake as a result of lipotoxicity [99]. OEA's hyperphagic actions are mediated by GPR119, resulting in an increase in cyclic adenosine monophosphate and adenylate cyclase, which is believed to occur through G_*α*s_ coupling [23]. Furthermore activation of PPAR*α* by OEA is believed to reduce inducible nitric oxide synthase (iNOS) gene expression, triggering a decrease in nitric oxide, which reduces vagal afferent stimulation and therefore appetite [23]. Both oleic acid and oleamide have been found to have similar actions in cultured microglial cells, through inhibition of lipopolysaccharide (LPS) induced iNOS activation, decreasing nitric oxide production as well as phosphorylation of Akt and the mitogen-activated protein kinase (MAPK) p38 [100, 101], which are both also GPCR signalling cascade components.

It has also been found that OEA increases FA release from adipocytes in a dose dependent manner and skeletal muscle FA uptake and oxidation without affecting glucose utilisation [102, 103]. Furthermore, OEA can reduce adipose tissue glucose uptake, mediated through the MAPK p38 and c-Jun N-terminal kinase (JNK) pathways [104], which inhibits the actions of AEA and 2-AG in adipose tissue and AEA-induced hyperphagia when both are administered intravenously [105]. This may explain the finding of an inverse correlation between adipose tissue MUFA content and degree of obesity (based on BMI and percentage body fat) and central adipose tissue distribution [106].

## 8. The Effect of Dietary Eicosapentaenoic and Docosahexaenoic Acid on Endocannabinoid Production

The role of dietary EPA and DHA in modulation of endocannabinoid synthesis has been extensively researched due to their ability to displace AA from phospholipid membranes and reduce its synthesis [107–109], resulting in greater production of eicosapentaenoyl ethanolamide (EPEA) and docosahexaenoyl ethanolamide (DHEA) (from the precursors EPA and DHA) [109]. While EPEA and DHEA do not appear to directly affect appetite, they have been demonstrated to decrease mouse adipocyte interleukin-6 and monocyte chemotactic protein-1 production, indicating anti-inflammatory properties [110].

Treatment of cultured mouse adipocytes with EPA/DHA in combination with different free FA found that DHA was able to counteract the conversion of AA to AEA and importantly was also able to stop the transfer of AA to the *sn*-1 position of phospholipids, from which AA can be converted to AEA [111]. Supplementation studies in both humans and animals have found that EPA/DHA decrease 2-AG [112, 113] and AEA [109, 113, 114] levels in obese subjects with a reduction in plasma n-3/n-6 ratio [112, 114] and a decrease in NAPE-PLD, FAAH, and CB_2_ mRNA expression [107], contributing to decreased receptor stimulation. Animal and human studies have also found that DHA/EPA supplementation results in a decrease in brain 2-AG levels [115], body mass [116] and prevents the development of obesity [117] and further weight gain in mouse models [118]. This may be due to an increase in *β*-oxidation [119] and a decrease in SREBP-1c [120], as well as the reduction in AEA and 2-AG production decreasing cannabinoid receptor stimulation and therefore appetite and food intake. Also possibly contributing to this is DHA/EPA increasing whole body insulin sensitivity by inhibiting LPS induced phosphorylation of JNK and nuclear factor kappa-B degradation and increasing Akt phosphorylation and glucose transporter type 4 translocation, via a GPR120 dependent pathway [24]. 

## 9. The Effect of Dietary Linoleic Acid on Endocannabinoid Production

Linoleic acid has been found to modulate endocannabinoid synthesis due to its ability to be converted to AA by the human body [31], although the effect of dietary linoleic acid on human endocannabinoid synthesis has not been investigated. This is a pertinent area of research due to the rapid increase in linoleic acid content in the Western diet as a result of a shift to plant-derived fats and the greater use of soy and corn oils in food production and manufacturing [29, 30]. These dietary changes have resulted in a shift in the n-3 to n-6 FA ratio, as reviewed by Simopoulos [121], with more than 84% of PUFA fats consumed in the USA being in the form of the AEA precursor linoleic acid [122]. High linoleic acid diets promote obesity in both animals and humans [123, 124] and are correlated with increased fasting blood glucose, fasting insulin [125], and insulin resistance [126] in humans, making this an important area of further research.

A study by Alvheim et al. (2012) replicated the Western diet linoleic acid increase in mouse feed, showing that increasing energy from linoleic acid from 1% to 8% in a diet with 60% of energy from lipids caused an increase in AA in the liver and red blood cells. This resulted in a subsequent 3-fold increase in both 2-AG and AEA and increased food intake, plasma leptin, and adiposity, possibly as a result of increased cannabinoid receptor activation; however, receptor expression and activation were not investigated in this study [26]. These changes were abolished with the addition of 1% n-3 PUFA to the 8% diet (resulting in levels comparable to those of the 1% linoleic acid diet), again demonstrating the ability of n-3 PUFA to displace AA and decrease endocannabinoid production [26]. A further study by the same researchers found that substituting fish oil with soy oil in salmon feed increased linoleic acid, AA, AEA, and 2-AG and decreased DHA and EPA in the salmon flesh and increased fat accumulation in the liver [127]. These fish were then fed to mice which resulted in an increased liver content of linoleic acid, AA, AEA, and 2-AG and decreased DHA and EPA, accompanied by weight gain and adipose tissue inflammation when compared to control fed animals [127]. This effectively demonstrated how changes in the linoleic acid content of animals produced for consumption can negatively affect the end consumer. Similarly, a study by Matias et al. (2008) using mice fed high MUFA and high PUFA diets for a 14-week period found that the high linoleic PUFA diet increased muscle 2-AG levels and induced obesity and hyperglycaemia (with significantly greater blood glucose concentrations than the MUFA diet) [128] indicating endocannabinoid system overactivity. Recently Dipatrizio et al. (2013) found that 30 minutes of oral exposure (through sham feeding) to linoleic acid resulted in an increase in both 2-AG and AEA in rat jejunums, which also triggered the rats to develop a preference towards fats with a high linoleic acid content, which did not occur when the animals were pre-treated with the CB_1_ agonists AM6546 and URB447 [77]. 

## 10. Conclusion

The manipulation of dietary FA has shown positive results in regard to endocannabinoid modulation and decreased cannabinoid receptor activity, although the majority of studies have been conducted in animals. These studies, however, have shown that both acute and sustained dietary FA modification is capable of modulating endocannabinoid production and therefore cannabinoid receptor activity and due to their role in appetite, affecting energy intake and therefore body weight. With obesity rates still escalating in prevalence and dietary guidelines emphasising a shift towards plant based fats, further research in this area is essential for the development of public health messages directed towards prevention and treatment of overweight and obesity and their related comorbidities.
